# Bilateral Luxatio Erecta Humeri With Acute Anterior-inferior Re-dislocation

**DOI:** 10.5811/cpcem.2019.9.44205

**Published:** 2019-11-19

**Authors:** Adam Kessler, Jacob Hinkley, David Houserman, Jacob Lytle, Michael Sorscher

**Affiliations:** * Ascension Genesys Hospital, Department of Orthopedics, Grand Blanc, Michigan; † Des Moines University, Des Moines, Iowa

## Abstract

Luxatio erecta is a description for a specific and rare type of shoulder dislocation where the humeral head dislocates directly inferior. This rare form of glenohumeral dislocation accounts for only 0.5% of shoulder dislocations. It is even less common for both shoulders to be bilaterally dislocated inferiorly with the characteristic “hands up” posture. A limited number of these bilateral occurrences are described in the literature to date and most have been from higher energy trauma. We have described a low energy case of bilateral luxatio erecta and the reduction method used and the continued instability following successful reduction under procedural anesthesia.

## INTRODUCTION

Luxatio erecta, meaning “to place upward” in Latin, is a term coined by Dr. Middeldorpf and Dr. Scharm in 1859 to describe the unique presentation of traumatic inferior dislocation of the glenohumeral joint.^1^ Patients classically present with the affected extremity held overhead in fixed abduction, with slight flexion of the elbow and pronation of the forearm, creating a so-called “hands up” posture.^2^ This is a rare diagnosis, accounting for 0.5% of shoulder dislocations, with only a total of 30 cases of bilateral luxatio erecta humeri (LEH) reported in the literature to date.^3^

The shoulder is inherently an unstable joint, with the large humeral head articulating with the smaller glenoid fossa. A combination of static and dynamic stabilizers contributes to the location of the humeral head in the glenoid socket. Injuries to either the static or dynamic stabilizers of the joint predispose to dislocation.^4^ By far, shoulder dislocations most commonly occur anteriorly, accounting for 95–97% of shoulder dislocations.^4^ Here, we present one such patient who presented with bilateral LEH after a ground-level fall.

## CASE REPORT

**Image 1:**
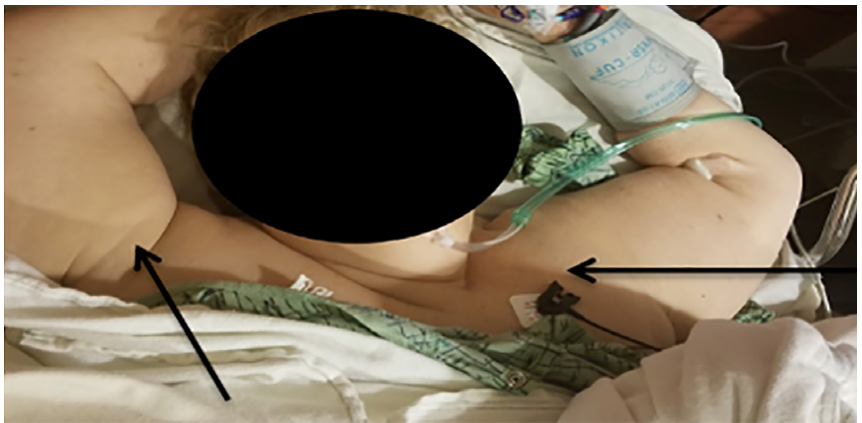
Clinical photograph of patient’s bilateral humeri held in abduction with forearms in pronation. Black arrows pointing to shoulders that are abducted and externally rotated.

**Image 2:**
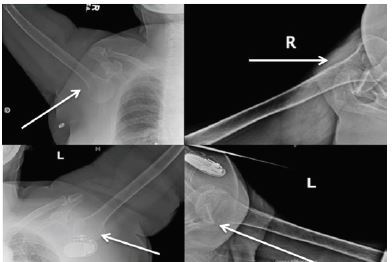
Radiographs demonstrating bilateral inferior shoulder dislocations without fracture. White arrows pointing to humeral head of bilateral shoulders, which is directly inferior to the glenoid.

We present a case of a 67-year-old female who was recently treated with a decompression and 10th thoracic to second lumbar fusion secondary to formation of an epidural hematoma from a 12th thoracic vertebra fracture (type unknown). While in a physical therapy session the patient suffered a fall forward, trying to break her fall with her arms outstretched above her head. After the fall the patient’s arms were stuck in full abduction and pronation and she was in significant pain. The patient’s presenting position is displayed in Image 1. She arrived in the emergency department where X-rays were taken and demonstrated bilateral inferior shoulder dislocations, LEH (Image 2).

Orthopedics was consulted to evaluate and treat. Upon evaluation, the patient was distally neurovascularly intact with 2/4 radial pulses bilaterally, sensation intact to light touch about the fifth cervical to first thoracic dermatomes, and motor function was intact in all peripheral motor groups of the upper extremities. The emergency physician provided sedation with closed reduction performed by the orthopedic service. The right shoulder was reduced using traction through the humerus through a flexed elbow to control the limb, and the opposite hand was used to place superior pressure on the humeral head through the axilla to guide the head into the glenoid. Slight external rotation and adduction was added as the head cleared the glenoid. Attention was then turned to the left shoulder, which was reduced, in a similar fashion; however, this shoulder was converted from an inferior to anterior dislocation using pressure in the axilla and slight external rotation.

**Image 3:**
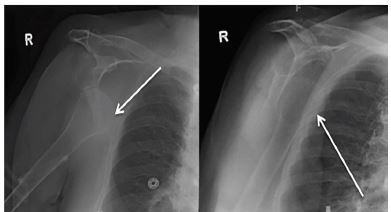
Radiographs demonstrating acute anterior-inferior right shoulder dislocation. White arrows pointing to the humeral head, which is anterior and inferior to the glenoid.

Following this, traction through the humerus external rotation, and lateral pressure on the humeral head yielded a successful relocation of the glenohumeral joint. After reduction the patient remained neurovascularly intact bilaterally. The patient was placed into bilateral shoulder slings, advised to avoid active shoulder range of motion, and admitted to the hospital for placement. Three days after the patient’s admission she adjusted a continuous positive airway pressure (CPAP) mask with her right arm dislocating anteroinferiorly (Image 3).

She was again noted to be distally neurovascularly intact. Sedation was performed by the anesthesia department, and the orthopedic service then performed closed reduction. The patient remained neurovascularly intact after reduction. She has not had another instability event to date.

## DISCUSSION

LEH is an extremely uncommon diagnosis, making it worthwhile to report. Our case is unique in that the patient experienced a subsequent unilateral non-traumatic anteroinferior shoulder dislocation within 48 hours of bilateral LEH reduction, which, to our knowledge, has never been described before in the literature on LEH.

**CPC-EM Capsule**

What do we already know about this clinical entity?

Luxatio erecta is a direct inferior shoulder dislocation accounting for only 0.5% of all shoulder dislocations and associated with higher rates of neurovascular injury.

What makes this presentation of disease reportable?

The presentation is a bilateral occurrence with a low energy mechanism followed by repeat instability which is rare in dislocations occurring in this age group.

What is the major learning point?

Despite low energy mechanism there was repeat instability. It is important to distinguish this presentation from routine simple shoulder dislocations.

How might this improve emergency medicine practice?

Recognizing this rare presentation may prompt earlier orthopedic consultation and follow-up. This may expedite treatment for continued instability.

The shoulder is inherently an unstable joint, with the large humeral head articulating with the smaller glenoid fossa. At any given moment only 25–30% of the humeral head surface is in direct contact with the glenoid surface.^5^ Joint congruity is maintained by both static and dynamic stabilizers. The glenoid labrum is a cartilaginous ring that acts as a static stabilizer to the joint, deepening the glenoid by 50%.^6^ Other static joint stabilizers include the glenohumeral ligaments, the articular congruity and version of the glenohumeral interface, and the negative intra-articular pressure created at the joint surface. The labrum combines with the joint capsule and ligaments to provide the remainder of static stability, deepening the articulation and serving as a capsuloligamentous attachment site.

These structures tighten with motion of the arm and are most functional at the extremes of motion. The coracohumeral and superior glenohumeral ligament act to prevent inferior translation with the arm adducted and posterior translation when forward flexed and internally rotated. The middle glenohumeral ligament is absent in 8–30% of people. Traveling from the supraglenoid tubercle and superior labrum to the medial aspect of the lesser tuberosity it prevents anterior translation in 60–90° of abduction and from inferior translation in adduction. The inferior glenohumeral ligament (IGHL) is the thickest and most consistent. Originating from the anterior inferior portions of the labrum to the lesser tuberosity of the humerus. The IGHL is mainly responsible for preventing anterior translation in abduction and external rotation.^5^ The rotator cuff musculature, long head of the biceps, and periscapular muscles act as dynamic stabilizers, providing stability throughout range of motion of the joint. Injuries to either the static or dynamic stabilizers of the joint predispose to dislocation, the most common direction being anterior in 95–97% of cases.^4^

Two main mechanisms of injury account for LEH, which are exclusively traumatic in nature.^7^ Commonly, an inferior force vector is applied on a fully abducted extremity, which disrupts the weaker inferior ligamentous complex allowing the humeral head to disengage the glenoid and dislocate inferiorly. Alternatively, hyperabduction injury can cause levering of the proximal humerus off of the acromion causing an inferior dislocation, usually from grasping an immobile object while falling to the ground. Our patient fell forward from a standing height while using her four-wheeled walker. It is unclear if she tried to catch herself on the walker with both hands while falling. However, the likely cause was bilateral hyperabduction moment leading to her dislocation. Nambiar et al. evaluated all of the published case reports regarding unilateral or bilateral LEH, and found that falls accounted for 45% of all cases, where falls from standing height accounted for 12% of all cases.^8^

Reduction techniques for LEH have been described in the literature with the most common reduction technique being overhead traction.^7^,^9^ This method involves bringing the patient’s arm into full abduction and, with an assistant to provide counter traction, physician applies upward directed force. Traction is maintained as the arm is slowly brought down into adduction. This method requires significant force to overwhelm the shoulder musculature and often requires conscious sedation.^7^,^9^,^10^

Nho and colleagues have described a separate two-part reduction maneuver that they successfully used in two patients described in their case report. In this technique the patient is placed supine and the physician stands on the affected side next to the head of the patient. The pushing hand should be placed on the lateral aspect of the midshaft humerus while the pulling hand is positioned over the medial epicondyle. The push hand manipulates the humeral head to an anterior position relative to the glenoid while the pull hand gives gentle superior directed force moving the head to anterior. The second step can be various methods for reduction of anterior dislocations including traction counter traction, or Nho’s preferred external rotation method. With patients arm completely adducted the push hand gives constant adduction force while the pull hand is relocated to the forearm to produce external rotation of the humerus until reduction is palpated.^9^

The surgeon should be aware of the well-known complications of LEH, which include associated fractures of the proximal humerus, acromion and clavicle, avulsion of the greater tuberosity, ligament and soft tissue injury such as rotator cuff tear and, less frequently, neurovascular injury. Ngam et al. performed a systematic review of five publications describing the magnetic resonance imaging features of LEH, and found that up to 75% of patients with traumatic LEH had concomitant rotator cuff tears. They observed that patients never sustained both a rotator cuff tear and a greater tuberosity avulsion, suggesting that avulsing the greater tuberosity spares the rotator cuff and vice-versa.^3^

Mallon et al. reviewed 80 cases of LEH and found that either a fracture of the greater tuberosity or rotator cuff tear were present in 80% of patients.^11^ Another characteristic finding in traumatic LEH is a Hill-Sachs-like impaction fracture at the posterosuperior humeral head, located more superior and lateral than classic Hill-Sachs lesions, from impacting the humeral head against the inferior glenoid.^7^ Computed tomography (CT) is the best imaging modality to evaluate for this Hill-Sachs-like lesion, or for radiographically occult fracture. CT was not obtained in our patient’s case because there was no clinical suspicion or radiographic evidence of fracture.

A lesser-reported complication of LEH is recurrent instability, and there is a paucity of literature reporting rates of re-dislocation with no reports of *anterior* instability and dislocation. Olds et al. performed a meta-analysis quantifying the recurrence rate and risk factors predicting re-dislocation after primary anterior shoulder dislocation. The study found that factors leading to increased risk of dislocation included age <40 years old had an odds ratio (OR) [13.46], male sex (OR [3.18]), and hyperlaxity in >1 joints (OR [2.68]).^12^ Our patient had none of the predictive risk factors for recurrent dislocation; there was no history of shoulder instability, hyperlaxity, and she was a female >40 years old. There was no radiographic evidence of acute fracture, bony Bankart lesion or Hill-Sachs-like deformity.

Given the bilateral nature of her injury and need to conduct activities of daily living, the patient was put in simple slings with strict instructions to avoid forward flexion and abduction as an alternative to bilateral shoulder immobilizers. Her re-dislocation occurred while she was reaching her hand over her mouth to remove a CPAP machine. The position of her arm was approximately forward flexed and abducted to 90 degrees with 45 degrees of external rotation normally within the physiologic range. However, it resulted in an anteroinferior dislocation in our patient showing the degree of instability she developed due to her initial LEH insult.

## CONCLUSION

LEH is an uncommon presentation for glenohumeral dislocation. This case is even more rare as it is bilateral with an unusual mechanism. We describe techniques for reduction as well as the inherent instability present in the geriatric population once the rotator cuff is disrupted. The instability is demonstrated in the limited inpatient follow-up we have with a subsequent anterior dislocation.
